# CfLec-3 from scallop: an entrance to non-self recognition mechanism of invertebrate C-type lectin

**DOI:** 10.1038/srep10068

**Published:** 2015-05-15

**Authors:** Jialong Yang, Mengmeng Huang, Huan Zhang, Lingling Wang, Hao Wang, Leilei Wang, Limei Qiu, Linsheng Song

**Affiliations:** 1 Key laboratory of Experimental Marine Biology, Institute of Oceanology, Chinese Academy of Sciences, Qingdao, China; 2 Dalian Ocean University, Dalian 116023, China; 3 University of Chinese Academy of Sciences, Beijing, China

## Abstract

A C-type lectin (CfLec-3) from *Chlamys farreri* with three carbohydrate-recognition domains (CRDs) was selected to dissect the possible mechanisms of PAMP binding and functional differentiation of invertebrate lectins. CfLec-3 distributed broadly, and its mRNA expression in hemocytes increased significantly after stimulations with LPS, PGN or β-glucan, but not poly(I:C). The recombinant CfLec-3 (rCfLec-3) could bind PAMPs and several microbes. rCfLec-3 mediated hemocytes phagocytosis against *Escherichia coli* and encapsulation towards agarose beads. Obvious functional differentiation occurred among the three CRDs, as CRD1 exhibited higher activity to bind PAMPs, while CRD2/3 were expert in promoting hemocyte mediated opsonisation. The tertiary structural differences were suspected to be associated with such functional differentiation. PAMP binding abilities of CfLec-3 were determined by Ca^2+^-binding site 2 motif. When Pro in this motif of each CRD was mutated into Ser, their PAMP binding abilities were deprived absolutely. rCRD2 acquired mannan binding capability when its EPD was replaced by EPN, but lost when EPN in rCRD3 was changed into EPD. The Pro in Ca^2+^-binding site 2 was indispensable for PAMPs binding, while Asn was determinant for specific binding to mannan. It shed new insight into PAMPs binding mechanism of invertebrate C-type lectins and their functional differentiation.

C-type lectins are a large and diverse class of carbohydrate-sensing receptors. They can recognize and bind to the terminal sugars on glycoproteins and glycolipids in a Ca^2+^-dependent manner, either as cell surface receptors for microbial carbohydrates or as soluble proteins existing in tissue fluids^1^,^2^. Recently, many members of this superfamily are proved to be widely involved in both innate and adaptive immune responses, and they can (1) serve as a pattern recognition receptor (PRR) for specific binding to pathogen-associated molecular patterns (PAMPs)^3^,^4^,^5^, (2) initiate and regulate innate/adaptive immune responses^6^,^7^,^8^, (3) trigger opsonization of pathogens^9^,^10^, and (4) interact with self-ligands to mediate cellular functions such as adhesion^11^,^12^. The pathogen recognition and opsonization mediated by C-type lectin are of particular interest in the field of innate immunology.

The protein-carbohydrate interaction mediated by C-type lectins is benefited from their carbohydrate-recognition domain (CRD)^13^,^14^, which is a compact structural module containing conserved residue motifs. According to the number of CRDs and the architecture of domain, vertebrate C-type lectins are divided into 17 subgroups, and most of the subgroups contain only one CRD except the macrophage mannose receptor group^15^. Even in the macrophage mannose receptor, only one CRD is carbohydrate-binding-related, and most of other CRDs do not contain conserved motifs in Ca^2+^-binding site 2^15^. Hence, the carbohydrate binding behavior of vertebrate C-type lectins is not associated with the number of CRD.

In the CRDs, the residues with carbonyl side chains involved in Ca^2+^ coordination in site 2 form two characteristic motifs to participate in carbohydrate binding directly together with the calcium atom. The two characteristic motifs, “EPN” (Glu-Pro-Asn) and “QPD” (Gln-Pro-Asp), are contributed by the long loop region and contain two residues with carbonyl side chains separated by a proline in *cis* conformation. The carbonyl side chains provide two Ca^2+^-coordination bonds, form hydrogen bonds with the carbohydrate and determine the binding specificity. The *cis*-proline is highly conserved to maintain the backbone conformation that brings the adjacent carbonyl side chains into the positions required for Ca^2+^ coordination. The two motifs in Ca^2+^-binding site 2 are associated with specificity of carbohydrate binding directly, where CRDs with an EPN motif bind mannose or similar sugar, while CRDs with the QPD motif bind galactose or similar sugar^15^.

Recently, a lot of C-type lectins have been identified and characterized from invertebrates, and some of them are multi-domain C-type lectins^16^,^17^,^18^. Their binding behavior and mechanism seem to be contradictory with that of vertebrate C-type lectins. In invertebrates, each CRD of multi-domain C-type lectin contains a Ca^2+^-binding site 2 to bind carbohydrate individually^18^,^19^, which is far different from that in macrophage mannose receptor. Meanwhile, the motifs in Ca^2+^-binding site 2 of invertebrate CRDs are diverse. Besides the “EPN” and “QPD” existing in vertebrate C-type lectins, other motifs such as “EPD” (Glu-Pro-Asp), “QPN” (Gln-Pro-Asn) and “YPT” (Tyr-Pro-Thr) have also been identified from invertebrates^20^,^21^. Moreover, the CRD with an “EPN” motif from cotton bollworm C-type lectin has been found to bind mannose and galactose synchronously^19^. However, the functional assignment of each CRD in invertebrate multi-domain C-type lectins, and their binding specificity determined by the motifs or amino acids are still far from well understood.

In our previous study, a C-type lectin (CfLec-3) with three tandem CRDs was identified from *Chlamys farreri*, and its involvement in the immune response against *Vibrio anguillarum* has been characterized^21^. In the present study, the three CRDs in CfLec-3 were investigated comparatively by site-directed mutagenesis to reveal their functional differentiation and the mechanism of PAMP binding specificity, as well as their roles in the innate immunity.

## Results

### The broad distribution of CfLec-3 and its response to bacterial PAMPs stimulations

**Figure 1:**
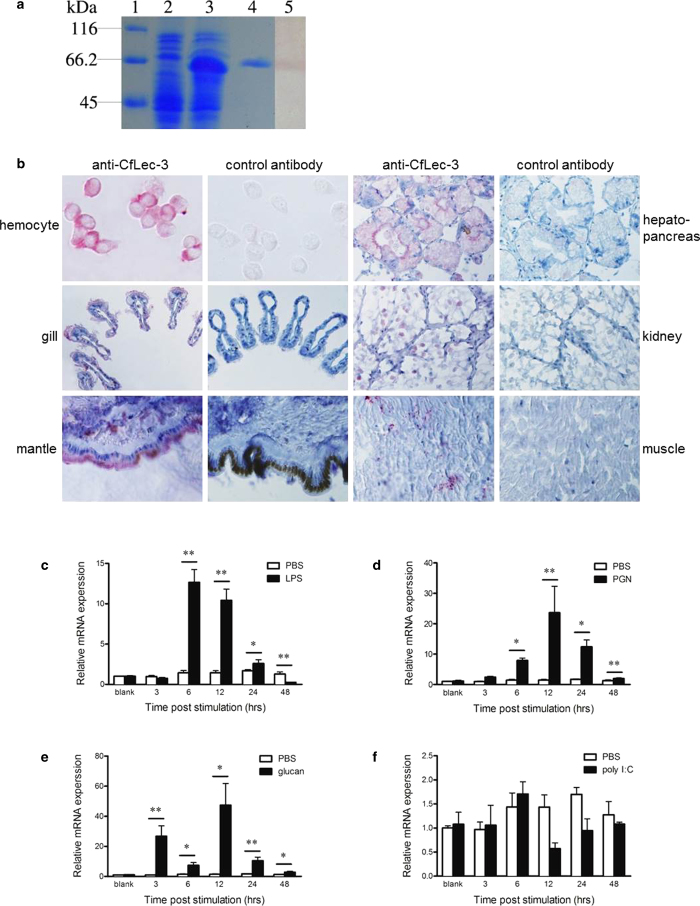
**a**, SDS-PAGE and Western-blot analysis of rCfLec-3. Lane 1: protein molecular standard; lane 2: negative control (without induction); lane 3: induced rCfLec-3; lane 4: purified rCfLec-3; lane 5: Western-blot of rCfLec-3 with anti-CfLec-3 antibody. **b**, Localization of endogenous CfLec-3 in different tissues by immunohistochemistry. CfLec-3 was detected by anti-CfLec-3 antibody, and stained in red. The tissues were counterstained with haematoxylin (blue). **c-f**, Temporal expression of CfLec-3 mRNA relative to β-actin was analyzed by realtime PCR in scallop hemocytes after LPS (**c**), PGN (**d**), β-glucan (**e**), poly I:C (**f**) and PBS challenge (**c, d, e, f**) for 3, 6, 12, 24 and 48 h. The values are shown as mean ± SE (N = 4), student’s *t*-test, error-bars represent biological repeats. (*: *P* < 0.05, **: *P* < 0.01).

C-type lectin plays crucial roles in both adaptive immunity and innate immunity to defense against pathogen infection^22^,^23^,^24^. Considering the large number of bacteria in their aquatic environment, marine mollusks employed amount of C-type lectins in almost all the tissues to protect themselves from continuous threat inflicted by the pathogens^25^,^26^,^27^,^28^,^29^. In the previous study, the mRNA transcripts of CfLec-3 were detected to be expressed universally in scallop tissues^30^. In the present study, the distribution of CfLec-3 protein was measured in order to further dissect its potential functions. The recombinant protein of CfLec-3 (rCfLec-3) and its polyclonal antibody were prepared according to the method reported previously^31^, and the antibody was proved to interact with CfLec-3 specifically (Fig. 1a). Be coinciding with our previous result about its mRNA expression pattern, the endogenous CfLec-3 localized in all the examined tissues including hepatopancreas, gill, kidney, mantle and muscle (Fig. 1b). Interestingly, CfLec-3 could also be observed on the surface of scallop hemocytes (Fig. 1b) although it was predicted to be a secreted protein, which was in accordance with another C-type lectin (CfLec-1) in *C. farreri*^31^, suggesting its potential roles in opsonizations. No positive signal was observed in all tissues from negative control group (Fig. 1b).

One hallmark of PRRs is their induced expression towards microbe or PAMP stimulations, and different isoforms of C-type lectins of *C. farreri* exhibited diverse expression profiles in response to the stimulations^28^,^29^,^31^,^32^. In the present study, four typical PAMPs including LPS, PGN, glucan and poly I:C were used to stimulate scallops and real-time RT-PCR was performed to monitor the mRNA expression of CfLec-3 transcripts in the hemocytes (Fig. 1c-f). In the LPS and PGN stimulated group, the mRNA expression of CfLec-3 was significantly (*P*<0.01) up-regulated, reached the maximal level (8.8-fold and 16.5-fold ) at 6 h and 12 h, respectively, and then decreased gradually to the origin level at 48 h post stimulation (Fig. 1c,d). The mRNA expression of CfLec-3 was up-regulated post β-glucan stimulation, and its expression level was 27.6, 5.1, 33.0 and 6.1-fold compared with PBS group at 3, 6, 12 and 24 h, respectively (Fig. 1e). However, there was no significant fluctuation of CfLec-3 expression post the stimulation of Poly I: C (*P*>0.05) (Fig. 1f). In the control group, there was no significant change of CfLec-3 expression during the whole experiment after PBS injection (Fig. 1c-f). These results suggested that CfLec-3 was inducible in response to stimulation of bacterial but not viral PAMPs.

### The PAMP binding activities of CfLec-3

**Figure 2:**
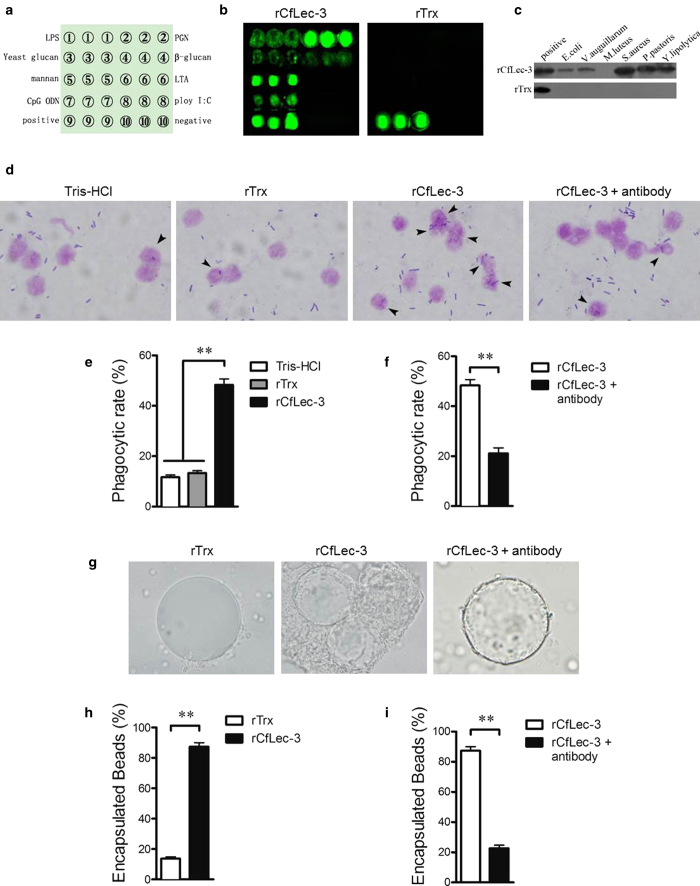
PAMPs binding assays of rCfLec-3 with microarrays. **a**, Sample order. **b**, PAMPs binding spectrum of rCfLec-3 and rTrx. **c**, The microbe binding spectrum of rCfLec-3 was revealed by Western-blot. Purified proteins rCflec-3 or rTrx was used as positive control. **d**, Phagocytosis enhanced by rCfLec-3. **e-f**, The Phagocytic rate (PR) of samples. Two hundred hemocytes on each slide were counted. **g**, rCfLec-3 promoted encapsulation of scallop hemocytes. Nickel agarose beads coated with rCfLec-3 or rTrx was incubated with scallop hemocytes, and encapsulation of the protein-coated beads was observed by microscopy after 6 h incubation. **h-i**, The percentage of encapsulated beads. The columns represent the mean of three individual counts ± S. E. M, student’s *t*-test, error-bars represent biological repeats, (*: *P* < 0.05, **: *P* < 0.01).

Since CfLec-3 mRNA could be induced by bacterial PAMPs, PAMPs binding assay was performed by the method of PAMP microarrays^31^ to find if it could bind these PAMPs directly. The binding activities of CfLec-3 to ten samples, including LPS, PGN, yeast glucan, β-1,3-glucan, mannan, lipoteichoic acids, CpG ODN, poly I:C, rabbit anti-rat IgG (positive control) and PBS-glycerol (negative control) were determined by the fluorescence signal (Table 1, Fig. 2a). rCfLec-3 bound obviously to LPS, PGN, yeast glucan, β-1, 3-glucan, mannan and CpG ODN with intense signal value, but not to lipoteichoic acids and poly I:C (Fig. 2b). As a control, rTrx did not bind any PAMP used in this experiment (Fig. 2b). Compared with previously identified C-type lectins in invertebrates, CfLec-3 exhibited definitely broad PAMPs binding capacity^33^,^34^,^35^.

### The enhanced phagocytosis mediated by CfLec-3

After PAMPs recognition and binding, PRRs could initiate multiply immune responses against pathogen. In order to further confirm the interaction between CfLec-3 and microorganism, rCfLec-3 was firstly incubated with Gram-negative, Gram-positive bacteria and fungi, and then analyzed by Western-blot assay. A clear band specifically for CfLec-3 was detected in the *Escherichia coli*, *Vibrio anguillarum*, *Staphylococcus aureus*, *Pichia pastoris* GS115 and *Yarrowia lipolytica* (Fig. 2c) group, respectively, while no band was detected in *Micrococcus luteus* (Fig. 2c) group. No obvious signal was observed in the rTrx negative control (Fig. 2c, bottom).

Phagocytosis assay was then performed to investigate whether CfLec-3 could enhance the immune response mediated by hemocytes. rCfLec-3 could significantly (*P*<0.01) enhance the phagocytosis of scallop hemocytes against *E. coli* (Fig. 2d). The phagocytic rate of scallop hemocytes treated with rCfLec-3, Tris-HCl and rTrx was 48.3%, 15.0% and 16.7%, respectively (Fig. 2e). Anti-CfLec-3 antibody was used to block binding of CfLec-3 to the hemocytes to confirm the enhanced phagocytosis was due to CfLec-3. When the anti-CfLec-3 antibody was added to the suspension of rCfLec-3 and hemocytes, the phagocytic rate was decreased to 21.1% (Fig. 2d,f), which indicated the phagocytosis of hemocytes was specifically enhanced by rCfLec-3.

### The encapsulation promoted by CfLec-3 *in vitro*

**Figure 3:**
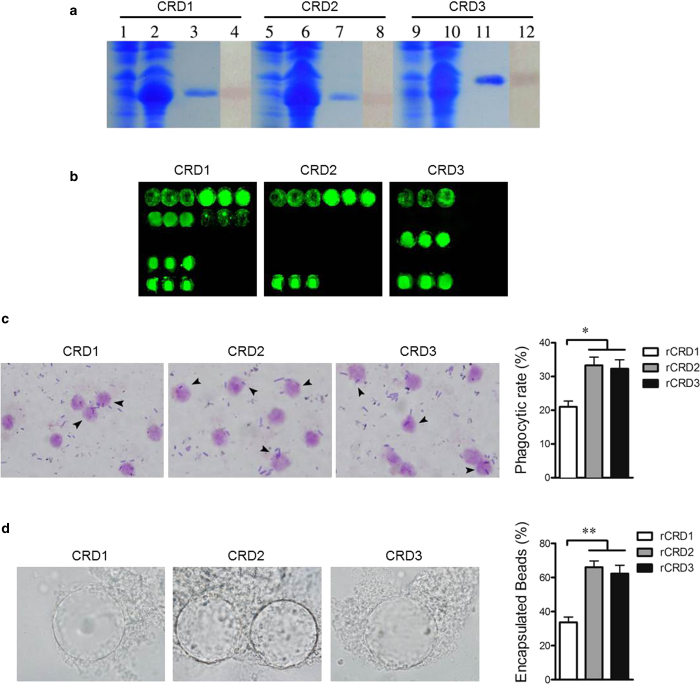
**a**, SDS-PAGE and Western-blot analysis of rCRD1, rCRD2, rCRD3. Lane 1, 5, 9: negative control of rCRD1, rCRD2 and rCRD3 (without induction); lane 2, 6, 10: induced rCRD1, rCRD2 and rCRD3; lane 3, 7, 11: the purified rCRD1, rCRD2 and rCRD3; lane 4, 8, 12: the Western-blot analysis of rCRD1, rCRD2 and rCRD3. **b**, PAMPs binding assays of rCRD1, rCRD2 and rCRD3 with microarrays. **c**, Phagocytosis enhanced by rCRD1, rCRD2 and rCRD3. **d**: rCRD1, rCRD2 and rCRD3 promote encapsulation of scallop hemocytes. The columns represent the mean of three individual counts ± S. E. M, student’s *t*-test, error-bars represent biological repeats (*: *P* < 0.05, **: *P* < 0.01).

Another opsonizations mediated by hemocytes was the encapsulation reaction against parasites^36^,^37^,^38^. To investigate whether CfLec-3 could promote encapsulation of hemocytes, *in vitro* encapsulation assay was performed using rCfLec-3 coated Ni-NTA agarose beads according to previous reports^39^. Compared with rTrx control, rCfLec-3 could enhance the encapsulation significantly (*P*<0.01) (Fig. 2g). Most of the beads (87.3%) coated with rCfLec-3 were encapsulated by scallop hemocytes in 6 h, while only a few beads (13.7%) coated with rTrx were encapsulated (Fig. 3h). Anti-CfLec-3 antibody was also used in order to determine the specific contribution of CfLec-3 to the augment of encapsulation. Effective blockage of encapsulation was detected after the lectin-coated beads were pre-incubated with CfLec-3 antibody (Fig. 3g,i), and the encapsulated beads decreased to 23%, suggesting CfLec-3 could indeed promote the encapsulation mediated by hemocytes.

### The functional differentiation of three CRDs in CfLec-3

Since there were three tandem CRDs identified in CfLec-3, they were recombinantly expressed and analyzed to determine their contributions to the immune responses mediated by CfLec-3. The recombinant proteins of three individual CRD were prepared, and all the three proteins could be specifically recognized by anti-rCfLec-3 antibody (Fig. 3a). By using PAMPs microarray, rCRD1 was found to bind LPS, PGN, yeast glucan, β-1,3-glucan and CpG ODN (Fig. 3b). In contrast, rCRD2 bound LPS and PGN (Fig. 3b), while rCRD3 bound LPS and mannan (Fig. 3b). These results suggested that CRD1, but not CRD2 and CRD3, contributed more to the PAMPs binding ability of CfLec-3. Phagocytosis and *in vitro* encapsulation were also investigated to understand the roles of individual CRD in immune responses. The phagocytic rate (Fig. 3c) and encapsulation rate (Fig. 3d) in CRD2 and CRD3 groups were higher than that in CRD1 group, indicating CRD2 and CRD3 were the key contributors for opsonizations mediated by hemocytes. All these results suggested that there was an obvious functional differentiation among the three CRDs in CfLec-3, and CRD1 was responsible for PAMPs recognition, while CRD2 and CRD3 were in charge of opsonization.

### The tertiary structures of the three CRDs in CfLec-3

**Figure 4:**
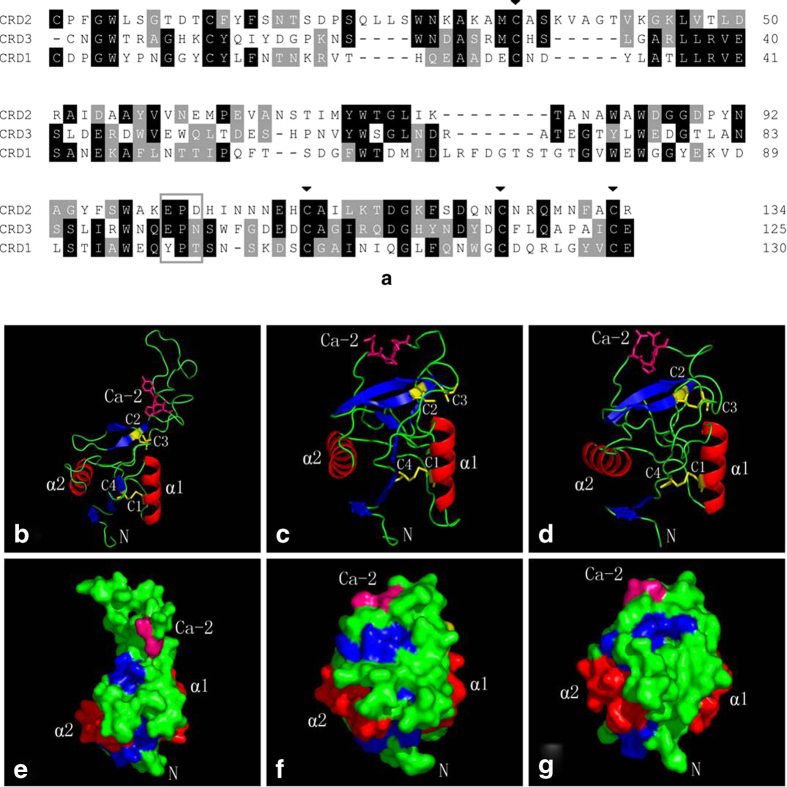
Sequence and structure analysis of three CRDs. **a**, Multiple sequence alignment of three CRDs. Amino acid residues that are conserved in at least 50%. Conserved cysteine residues involved in the formation of disulfide bridges were marked with ▼. The letters in box indicate the motif for Ca^2+^-binding site. **b-g**, Predicted tertiary structure of three CRDs. **b** and **e**, CRD1; **c** and **f**, CRD2; **d** and **g**, CRD3.

Sequence and structure analysis was performed to clarify the potential mechanism for the functional differentiated of three CRDs in CfLec-3. Multiple sequence alignment was developed by ClustalW to reveal the conserved and different sequences among the three CRDs (Fig. 4a). The four cysteine residues involved in the formation of internal disulfide bridges existed in all the CRDs. However, the motif in the Ca^2+^-binding site 2, which determined carbohydrate binding specificity, was different from each other. It was YPT in CRD1, EPD in CRD2 and EPN (Glu-Pro-Asp) in CRD3 (Fig. 4a).

The potential tertiary structures of three CRDs were established using the SWISS-MODEL prediction algorithm (Fig. 4b,c,d,e,f,g), and the E-values were 6.1E-13, 9.8E-18 and 4E-07, respectively. The structures of all the three CRDs adopted a typical double-loop structure consisting of two parts, a lower part composed of two conserved α-helices and two β-strands, and an upper part composed of two or four β-strands. Four conserved cysteines (C1-C4) formed two disulfide bridges at the bases of the loops. C1 and C4 linked the whole domain loop, while C2 and C3 linked the long loop region. CRD2 and CRD3 shared high similarity in tertiary structure (Fig. 4c,d,f,g), but the tertiary structure of CRD1 was far different from CRD2 and CRD3, especially in the position of Ca^2+^ binding site 2 (Fig. 4b,e). These results indicated that CRD2 and CRD3 shared the similar structure but they were different from CRD1, which might be associated with the differentiation of their functions.

### Motif in Ca^2+^-binding site 2 of CfLec-3 determined PAMPs binding specificity and capacity

**Figure 5:**
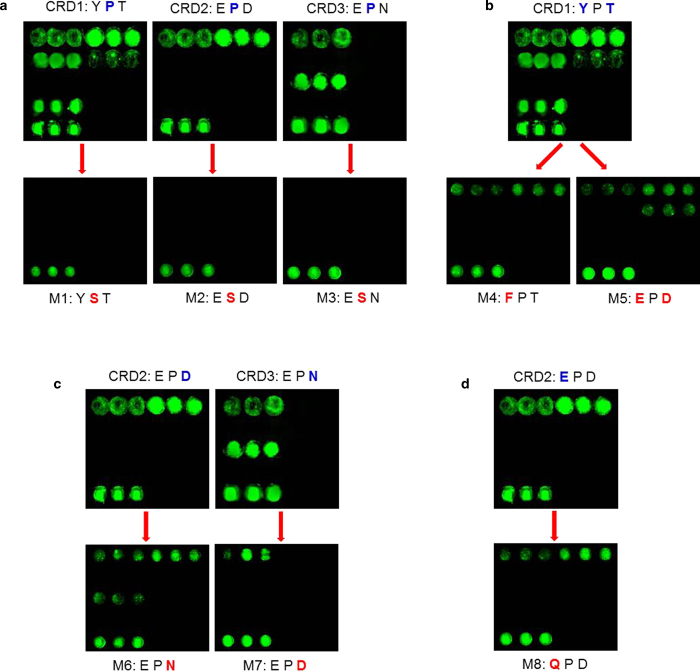
PAMPs binding assays of eight mutated rCRDs with microarrays. Samples were printed onto the glass slides as the order showed in Table 1. **a**, rCRDs with mutation 1, 2, 3 (M1, M2, M3). **b**, M4, M5. **c**, M6, M7. **d**, M8. The amino acids marked with blue were wild type amino acids; the ones marked with red were mutated amino acids.

To investigate the PAMPs binding mechanism of invertebrate C-type lectin, eight mutated CRD proteins were generated using site-directed mutagenesis targeting on the Ca^2+^ binding site 2 of the three CRDs in CfLec-3 (Table 2). PAMPs binding assay revealed that most of mutant CRDs exhibited different PAMPs binding spectrum from their original counterparts (Fig. 5). To date, the second amino acid of all the CRDs in known C-type lectins including the three CRDs of CfLec-3 is the unique P (Pro), which suggests that Pro is indispensable for PAMPs binding. In the present study, Pro in CRD1, CRD2 and CRD3 was replaced by Ser to generate three mutated protein M1, M2 and M3, whose Ca^2+^ binding site 2 was “YST” (Tyr-Ser-Thr), “ESD” (Glu-Ser-Asp) and “ESN” (Glu-Ser-Asn) respectively. All these three mutated proteins were deprived the ability of PAMPs binding and they could not bind all the tested PAMPs (Fig. 5a), suggesting the second amino acid Pro was indispensable for PAMPs binding ability of C-type lectins.

The “YPT” identified in CRD1 was a unique motif which had never been reported in all the known C-type lectins, and CRD1 was proved to be the key contributor of PAMPs binding in the present study. Two mutated CRD1, M4 and M5, were generated targeting on “YPT”, in which the motif “YPT” was mutated into “FPT” (Phe-Pro-Asp) and “EPD”, respectively, to investigate the mechanism of broadly PAMPs binding ability of CRD1. PAMPs binding assay revealed that M4 and M5 bound less PAMPs than wild type CRD1. M4 bound LPS and PGN (Fig. 5b), while M5 bound LPS, PGN and β-1, 3-glucan (Fig. 5b). These results indicated that “YPT” motif in Ca^2+^ binding site 2 of CRD1 determined broad PAMPs binding spectrum of CfLec-3.

It was interesting that there was only one amino acid different between CRD2 (EPD) and CRD3 (EPN) which determined the PAMPs binding abilities. To better understand the PAMPs binding mechanism mediated by motif in Ca^2+^ binding site 2, “EPD” in CRD2 was replaced by “EPN”, while “EPN” in CRD3 was mutated into “EPD”. When the “EPD” in CRD2 was replaced by “EPN”, the mutant protein (M6) acquired the mannan binding ability (Fig. 5c). When the “EPN” in CRD3 was mutated into “EPD” (M7), the mannan binding ability disappeared correspondingly (Fig. 5c). It directly suggested that the amino acid “Asn” in the motif of Ca^2+^-binding site 2 determined the binding specificity of lectin towards mannan. In contrast, when motif “EPD” in CRD2 was changed into “QPD” that was frequently observed in vertebrates’ CRDs, the mutant protein M8 did not exhibit distinct alteration in PAMPs binding profile (Fig. 5d).

### Phagocytosis but not encapsulation was associated with Ca^2+^-binding site 2 motif

**Figure 6:**
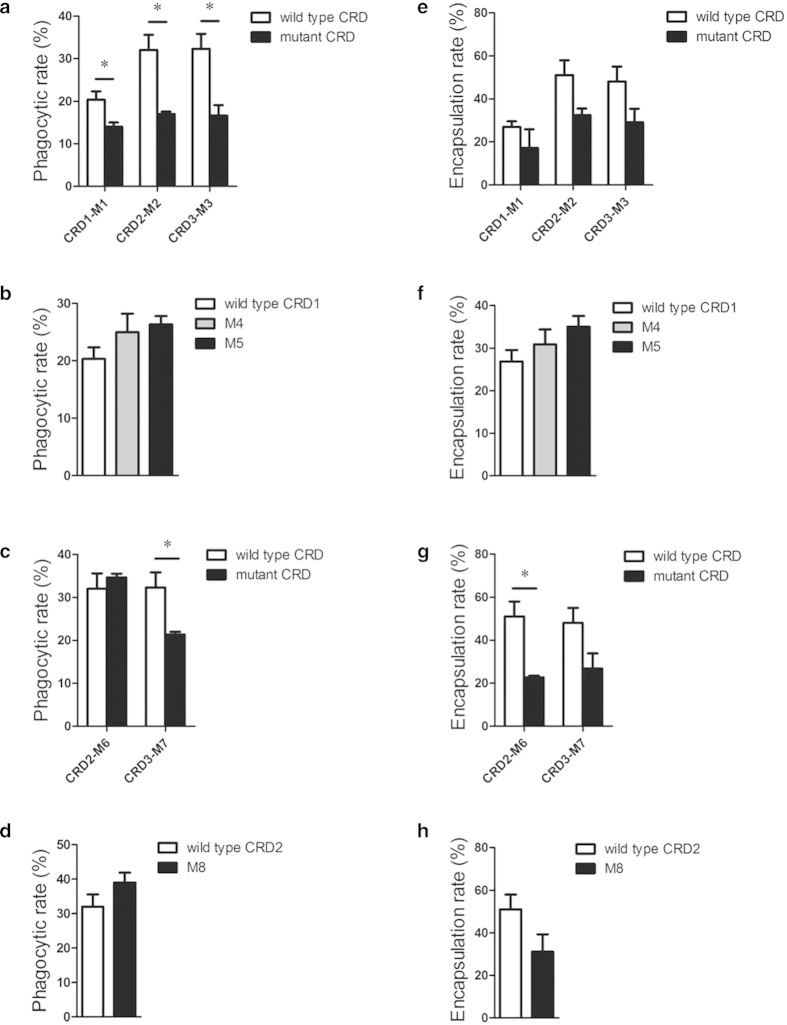
**a-d**, Phagocytosis enhanced by eight mutated rCRDs. **e-h**, Encapsulation enhanced by eight mutated rCRDs. The columns represent the mean of three individual counts ± S. E. M, student’s *t*-test, error-bars represent biological repeats (*: *P* < 0.05, **: *P* < 0.01).

The phagocytosis and encapsulation of hemocytes mediated by mutated CRDs were measured to find the association of motif in Ca^2+^-binding site 2 with immune responses. The phagocytosis rates of mutated CRDs M1, M2 and M3 were all lower compared with that of their wild type CRD counterparts (Fig. 6a), suggesting they would dysfunction from promoting phagocytosis. However, no obvious differences were found for the other five mutations, except M7 which slightly decreased its activity to enhance phagocytosis (Fig. 6b-d). On the other hand, *in vitro* encapsulation assay showed that most of mutations did not affect the abilities to promote encapsulation of hemocyte, except M6 whose encapsulation rate was relative lower than that of the wild type CRD2 (Fig. 6e-h). All the results indicated that phagocytosis but not encapsulation was associated with Ca^2+^-binding site 2 motif, especially the second amino acid “Pro”.

## Discussion

Among all the pattern recognition molecules, C-type lectins are identified as the indispensable ones for their diverse and prominent abilities in recognizing and binding pathogens. In the present study, CfLec-3 distributed in a wide range of tissues including hepatopancreas, gill, kidney, mantle and muscle of *C. farreri*, and its mRNA expression level in the hemocytes was up-regulated rapidly upon bacterial PAMPs stimulation. The wide distribution and the acute response against PAMPs stimulation of CfLec-3 mRNA suggested its important roles in immune defense of scallops against invading pathogens. In addition, though CfLec-3 was predicted to be a secreted protein with a signal peptide in its deduced amino acid sequence, it could bind to the surface of scallop hemocytes like other invertebrate C-type lectins^16^,^40^, indicating that it would be involved in the immune response mediated by hemocytes.

To date, the C-type lectins identified from invertebrates exhibited diverse PAMPs recognizing and binding spectrum. For instance, BmMBP from silkworm could bind LTA, PGN, and mannan, but not LPS^18^. AmphiCTL1 from amphioxus interacted with PGN and glucan, but not with LPS, LTA, and mannan^41^. In the current study, the mRNA expression of CfLec-3 was quickly up-regulated after stimulation of LPS, PGN and β-glucan, indicating CfLec-3 could recognize these PAMPs and be involved in innate defense as part of the acute-phase response against bacteria and fungi. However, the mRNA expression of CfLec-3 was not induced by the stimulation of poly I: C, suggesting it could not trigger the immune response against virus. rCfLec-3 could bind LPS, PGN, yeast glucan, β-1,3-glucan, mannan and CpG ODN, and its PAMPs binding spectrum was broader than those of any other known invertebrate C-type lectins^42^,^43^. So far, several C-type lectins have been identified from *C. farreri* in our previous studies, and they exhibited different PAMPs binding spectrum. For example, CfLec-1 bound LPS, PGN and mannose^31^, CfLec-2 bound LPS, PGN, mannose and zymosan^29^, and CfLec-4 bound LPS, PGN, glucan and mannose^28^. It seemed that different C-type lectin in *C. farreri* might mediate specific PAMPs recognition, and the superfamily of C-type lectin with specificity in recognition and complementarity provided a prominent immune defense network for scallop against invaders. Meanwhile, rCfLec-3 could bind *E. coli*, *V. auguillarum*, *S. aureus*, *P. pastoris* GS115 and *Y. lipolytica*, which accorded with its PAMP binding specificity. In order to reveal the contribution of each CRD to CfLec-3, the ligand binding assay was performed to determine the binding activity of each recombinant single CRD. rCRD1 could bind five kinds of PAMPs, while rCRD2 or rCRD3 could bind only two, indicating a dominant contribution of CRD1 for the broad PAMPs binding ability of CfLec-3, and the ligand spectrum of CfLec-3 was exactly the sum of its three CRDs.

Opsonins are host-derived proteins that can increase the efficiency of phagocytosis and diversify the functional repertoire of phagocytes^44^,^45^. Two archetypical opsonins, complement fragment C3bi which binds nonspecifically to the surface of foreign particles and immunoglobulin (Ig) which attaches to the target particle by recognizing specific surface epitopes, have been extensively characterized^45^. Very recently, several transmembrane C-type lectins, such as mannose receptor, Dectin-1 and DC-SIGN, have been found to play certain roles in opsonization by triggering the phagocytosis against invaders directly or indirectly^9^. Some invertebrate C-type lectins are found to adhere on the membrane of hemocytes to trigger the opsonization^40^. Phagocytosis and encapsulation are two generalized opsonization against bacteria and parasites in invertebrates, and they are both initiated by the binding of pathogen to phagocytes. In the present study, CfLec-3 was found to recognize and bind the pathogen component on cell surface, as well as to bind the surface of hemocytes. The multi-direction binding significantly enhanced phagocytosis against *E. coli* and encapsulation towards agarose beads. These results indicated that CfLec-3 was an opsonin-like molecule which could attach on the non-self agents and trigger the phagocytic and encapsulating abilities of hemocytes. Meanwhile, the three CRDs in CfLec-3 all exhibited the ability to induce opsonization of hemocytes, but CRD2 and CRD3 displayed more significant ability than that of CRD1 to trigger the opsonization, indicating that CRD2 and CRD3 were the main contributor of the opsonization triggered by CfLec-3. The results clearly suggested that the three CRDs in CfLec-3 experienced a process of functional differentiation, and CRD1 was responsible for pathogen recognition, while CRD2 and CRD3 were in charge of opsonization.

In the course of evolution, the functional differentiation of a protein has been becoming increasingly meticulous^42^. As the elementary unit, the functional domain embodies the essential information, and always determines the functions of the protein. In the present study, an obvious functional differentiation was found among the three CRDs in CfLec-3, and there should be some potential elements involved in such differentiation. Multiple sequence alignment revealed that the three CRDs shared significant sequence similarity, and the four cysteine residues involved in the formation of the internal disulfide bridges were well conserved in each CRD. However, the motif in the Ca^2+^-binding site 2 was different from each other. It was “YPT” in CRD1, “EPD” in CRD2, while “EPN” in CRD3. Among them, “EPN” was a typical motif in both vertebrate and invertebrate C-type lectins, and “EPD” was a common motif in invertebrate C-type lectin, but “YPT” was a unique motif of Ca^2+^-binding site 2 which had never been found in the previously identified C-type lectins. The sequence variation of CRDs might affect the function of C-type lectins, especially the carbohydrate binding ability. It has been reported that only a few difference of amino acids in CRDs will change the activity of C-type lectins^46^. In the present study, the tertiary structure of three CRDs in CfLec-3 was established, and the tertiary structures of CRD2 and CRD3 were similar even though there were obvious differences in the Ca^2+^-binding site 2. It was interesting that the tertiary structure of CRD1 was far different from that of CRD2 and CRD3, not only in the Ca^2+^-binding site 2, but also in all the upper part. To our knowledge, the tertiary structure of all the identified C-type lectins have always been well conserved even though their primary sequences were more or less different. The obvious difference between the tertiary structures of CRDs in CfLec-3 was considered to be responsible for their functional differentiation.

It has been accepted that C-type lectins bind carbohydrate in a specific manner, which is determined by the position of hydrogen bond donors and acceptors in Ca^2+^-binding site 2 of CRDs^4^. The CRDs with an “EPN” motif bind mannoses or similar sugar, whereas those with “QPD” motif bind galactose or similar sugar. However, accumulating evidences have demonstrated the diverse structure and multifunction of invertebrate C-type lectin, which is different from that of vertebrate C-type lectin. There is a common feature in all the CRDs identified in both vertebrate and invertebrate that the second amino acid in the motifs of Ca^2+^-binding site 2 is always “Pro”. Structurally, the side chain of “Pro” is fused back to the nitrogen of the backbone, and strongly associated with β-turns to fold the protein, which is essential to keep the stability and bind metal ion^47^. In the present study, eight mutants were constructed, and their PAMP binding abilities were examined in order to find the possible PAMP binding mechanism of CfLec-3. When the “Pro” in the motif “YPT” (CRD1), “EPD” (CRD2) and “EPN” (CRD3) was replaced by S (Ser), all the CRDs lost their ability to bind PAMPs absolutely. The results clearly indicated that the amino acid “Pro” in Ca^2+^-binding site 2 was indispensable for the PAMPs binding. The “YPT” identified in the present study was a unique motif which had never been reported in all the known C-type lectins, and CRD1 with “YPT” motif exhibited ability to bind LPS, PGN, yeast glucan, β-1,3-glucan and CpG ODN. When the “YPT” was mutated into “FPT” and “EPD”, respectively, the CRD1 mutant (M4 with FPT) only bound LPS and PGN, while M5 with EPD bound LPS, PGN and β-1, 3-glucan. Because the -OH group of the amino acid “Tyr” is able to donate or accept hydrogen bonds, the broad PAMPs binding ability of “YPT” motif is suspected to benefit from this amino acid, and it could ionize and participate in the acid-base reactions of many active sites to promote the Ca^2+^ and PAMPs binding. Interestingly, when the “EPD” in CRD2 was replaced by “EPN”, it acquired the mannan binding ability, while when the “EPN” in CRD3 was mutated into “EPD”, the mannan binding ability disappeared correspondingly. This fact directly suggested that the amino acid “Asn” in the motif of Ca^2+^-binding site 2 determined the binding specificity of lectin towards mannan. Additionally, the results also suggested that the second amino acid “Pro” in the Ca^2+^-binding site 2 was associated with phagocytosis but not encapsulation mediated by hemocytes, When “Pro” was mutated into “Ser”, all the three CRDs were deprived of their abilities to promote phagocytosis of hemocytes, but no obvious effect could be found to the encapsulation activities. Considering the mutated CRDs M1, M2 and M3 lost their PAMPs binding abilities completely, the phagocytosis against *E. coli* was suggested to be a response depending on PAMPs binding abilities, but encapsulation against non-living agarose beads was a PAMPs binding ability-independent manner. Our results significantly broadened the knowledge on the ligands binding mechanism of invertebrate C-type lectin and also illustrated their efficient role in modulating innate immune response against invaders.

## Conclusion

A C-type lectin (CfLec-3) with three carbohydrate-recognition domains (CRDs) from *Chlamys farreri* was significantly (*P*<0.01) up-regulated in hemocytes after PAMPs stimulation. rCfLec-3 mediated hemocytes phagocytosis against *E. coli* and encapsulation towards agarose beads. Every single rCRD could mediate opsonization individually, and the activity of rCRD2 and rCRD3 was significantly higher (*P*<0.01) than that of rCRD1. rCRD1 displayed the same PAMP binding patterns as rCfLec-3 which bound LPS, PGN, yeast glucan, β-1,3-glucan, mannan and CpG, while rCRD2 only bound LPS and PGN, and rCRD3 bound LPS and mannan. When Pro in Ca^2+^-binding site 2 motif of each CRD was mutated into Ser, their PAMPs binding ability was deprived absolutely. The rCRD2 mutant acquired mannan binding capability when motif EPD was replaced by EPN. The rCRD3 mutant lost mannan binding capability when EPN was changed into EPD. These results indicated there was functional differentiation among the CRDs, with CRD1 mainly responsible for pathogen recognition, while CRD2 and CRD3 in charge of opsonization. And Pro in Ca^2+^-binding site 2 was indispensable for PAMPs binding, while Asn was determinant for specific binding to mannan.

## Methods

### Ethics statement

Female Wistar rats were purchased from Qingdao Institute for the Control of Drug Products (Qingdao, China). All animal experiments were carried out following a protocol which had been approved by the Animal Care and Use Committee at Qingdao institute for the control of drug products with a permit number of SCXK (Shandong) 20090007. No surgery was performed in the present study, and all efforts were made to minimize suffering.

### Scallops, microbe and immune stimulation

Adult scallops *C. farreri* with an average 55 mm in shell length were collected from a farm in Qingdao, Shandong Province, China, and maintained in the aerated seawater at 15 °C for a week before experiments. The scallops used in the present study were followed the regulations of local and central government.

Bacteria *M. luteus*, *S. aureus* and *E. coli* were purchased from Microbial Culture Collection Center (Beijing, China) and *P. pastoris* GS115 was purchased from Invitrogen (CA, USA). *V. anguillarum* was kindly provided by Dr. Zhaolan Mo, and *Y. lipolytica* was kindly provided by Dr. Zhenming Chi.

The scallops were randomly divided into 6 groups and each group contained 40 individuals. The scallops in five groups received an intramuscular injection of 50 μL phosphate buffered saline (PBS, 0.14 M sodium chloride, 3 mM potassium chloride, 8 mM disodium hydrogenphosphate dodecahydrate, 1.5 mM potassium phosphate monobasic, pH 7.4), LPS from *E. coli* 0111:B4 (Sigma-Aldrich, 0.5 mg mL^−1^ in PBS), PGN from *S. aureus* (Sigma-Aldrich, 0.8 mg mL^−1^ in PBS), β-glucan from *Saccharomyces cerevisiae* (Sigma-Aldrich, 1.0 mg mL^−1^ in PBS), and Ploy I:C (Sigma-Aldrich, 1.0 mg mL^−1^ in PBS), respectively. The scallops treated with PBS were employed as control group, and the untreated scallops as blank group. After treatment, the scallops were returned to water tanks and 5 individuals were randomly sampled at 0, 3, 6, 12, 24 and 48 h post-injection. The hemolymphs were collected, and centrifuged at 500 × *g*, 4 °C for 10 min to harvest the hemocytes for RNA preparation.

### Preparation of antibodies and Western-Blot analysis

The cDNA fragments encoding the peptide of CfLec-3, CRD1, CRD2 and CRD3 were recombine into plasmid pET30a (for CfLec-3) or pET32a (for CRD1, CRD2 and CRD3), and the recombinant proteins were prepared, purified and refolded by the method described in previous report^30^. The renatured protein rCfLec-3 was immuned to 6-week old rats to acquire polyclonal antibody and the antiserum was purified as the method described by Cheng^48^. The specificity of the antibody was detected by Western-Blot analysis as previous description^31^.

### Immunohistochemistry detection of CfLec-3 protein in tissues

The slides of hemocytes were prepared as previously described^42^. The tissues of *C. farreri*, including gill, adductor muscle, hepatopancreas, mantle and kidney were fixed in Bouin’s fluid for 24 h and embedded in paraffin wax. Longitudinal sections (5 μm) were cut and mounted on slides, and then subjected to deparaffination in xylene and rehydration in diluted ethanol series. The hemocytes and tissue sections were incubated with 3% acetic acid for 20 min to eliminate endogenous alkaline phosphatase. Antigen retrieval was performed by heating the tissue sections in water bath for 10 min.

After 1 h incubating in PBS containing 3% BSA, the slides were covered with 20 μL antibody of rCfLec-3 (diluted 1:500 in PBS) and incubated at 37 °C for 1 h in a moisture chamber. After three times washing with PBS containing 0.05% Tween-20 (PBS-T), the slides were incubated at 37 °C for 1 h with 20 μL of biotinylated rabbit anti-rat IgG (H+L) (5 μg mL^−1^). Then the slides were incubated with 20 μL of alkaline phosphatase streptavidin (3 μg mL^−1^) at 37 °C for 1 h after strict washing. Finally, the reaction was developed with Histomark RED substrate for 10 min, slightly counterstained with haematoxylin and then mounted in buffered glycerin. Rats’ preimmune serum was used as negative control.

### Expression analysis of CfLec-3 post PAMPs stimulation

The cDNA template was synthesized by the method described by Huang^28^. One pair of gene-specific primer were used to amplify a CfLec-3 cDNA fragment of 222 bp, and two β-actin primers were used to amplified a 94-bp fragment as an internal control (Table S1). All the RT-PCR was performed and analyzed as previous description^49^, and the data were presented in terms of relative mRNA expressed as mean ± SE (N = 4). Differences were considered significant at *P* < 0.05 and extremely significant at *P* < 0.01.

### PAMPs binding assay

The PAMPs binding assay was performed by the method of PAMPs microarray according to the previous report^49^. rCfLec-3, rCRD1, rCRD2 and rCRD3 were added onto the microarrays, respectively, and the same dose of rTrx was set as a control. Twenty microliter of Cy3-conjugated anti-CfLec-3 antibody was added and incubated at 37 °C for 1 h. The images of the microarrays were obtained using a laser chipscanner at 532 nm, and analyzed with EcoscanCHS software to quantify florescence intensity.

### Microbes binding assay

Gram-positive bacteria (*M. luteus, and S. aureus*), Gram-negative bacteria (*E. coli* and *V. anguillarum*) and fungi (*P. pastoris* GS115 and *Y. lipolytica*) were used to test the binding spectrum of rCfLec-3 to microorganisms according to the method described by Lee^50^. rTrx was used as negative control.

### Phagocytosis assay

Phagocytosis assay was performed according to previous method with modification^42^. Briefly, hemolymph was collected from five scallops with equal volume of pre-chilled anticoagulant (Tris-HCl 50 mM; glucose 2%, NaCl 2%; EDTA 20 mM; pH 7.4), and then centrifuged at 800 g for 10 min to harvest the hemocytes (1 × 10^6^). After incubation with rCfLec-3, rCRD1, rCRD2, rCRD3, mutated CRDs and rTrx (100 μg mL^−1^) at 18 °C for 30 min, 5 μL *E. coli* (OD_600_ = 0.4) was added into each hemocytes suspension, and incubated at 18 °C for another 1 h. The phagocytic rate of hemocytes was measured using a light microscope as Phagocytic rate (PR) = (phagocytic hemocytes) / (total hemocytes) × 100%. To test whether phagocytosis was specifically enhanced by CfLec-3, 200 μL of rCfLec-3 (100 μg mL^−1^), the antibody of rCfLec-3 (5 μg) and hemocytes suspension (1 × 10^6^ cell) were incubated at 18 °C for 30 min, and then 5 μL *E. coli* (OD_600_ = 0.4) was added and incubated at 18 °C for another 1 h. The phagocytic rate of hemocytes was measured with the method as described above.

### *In vitro* encapsulation assay

*In vitro* encapsulation assay was performed according to previous report^51^. Briefly, Ni-NTA agarose beads (Qiagen) were equilibrated in TBS containing 5 mM CaCl_2_, and then incubated with renatured His-tagged rCfLec-3, rCRD1, rCRD2, rCRD3, mutated CRDs and rTrx in a 1.5 mL tube with shaking at 4 °C overnight, respectively. A 48-well cell culture plate (Costar) was coated with 1% agarose (Qiagen). The diluted hemocytes (about 10^6^ Cells mL^−1^) in Leibovitz L-15 medium (Sigma) were added to each well of the agarose-coated plate. After the hemocytes were allowed to settle down for at least 10 min, 1 μL (100-120 beads) of the protein-coated agarose beads was added to each well, and the plate was incubated at 18 °C for 6 h. The encapsulation of the agarose beads was observed under a light microscope. For each recombinant protein, the assay was performed in three different wells for statistical analysis. To test whether *in vitro* encapsulation was specifically incited by CfLec-3, 5 μL protein-coated beads were placed in a microcentrifuge tube, and 5 μg of rCfLec-3 antibody (in TBS, pH 7.5) was added and incubated at 4 °C overnight with shaking. Then the beads were washed with TBS and suspended in 5 μL TBS. *In vitro* encapsulation assay was performed as described above.

### Sequence and structure analysis and site-directed mutagenesis of three CRDs in CfLec-3

The ClustalW Multiple Alignment program ( http://www.ebi.ac.uk/clustalw/) was used to create the multiple sequence alignment. The presumed tertiary structure of the three CRDs from CfLec-3 were established by using the SWISS-MODEL prediction algorithm ( http://swissmodel.expasy.org/) and displayed by pymol version 0.97.

Site-directed mutagenesis of three CRDs in CfLec-3 was performed by using QuikChange® Lightning Site-Directed Mutagenesis Kit (Stratagene). Totally eight mutations were constructed targeting on the Ca^2+^ binding site 2 of three CRDs (Table 1) according to the instruction manual with the gene-specific primer (Table 2). Subsequently, the recombinant proteins of eight mutations were prepared, purified and renatured, and the PAMPs binding assay of the mutative proteins was performed as described above.

## Author Contributions

J.Y. participated in design of the study, performed all experiments and drafted the manuscript. M.H. carried out the microbe binding experiment, functional assay of mutated CRDs and drafted the manuscript. LW participated in the design and coordination and helped to draft the manuscript. H.Z. and H.W. participated in the recombination of proteins. L.W. participated in the phagocytosis enhancement of proteins. L.Q. helped to draft the manuscript. L.S. designed the study, drafted and revised the manuscript.

## Additional Information

**How to cite this article**: Yang, J. *et al*. CfLec-3 from scallop: an entrance to non-self recognition mechanism of invertebrate C-type lectin. *Sci. Rep.* **5**, 10068; doi: 10.1038/srep10068 (2015).

## Supplementary Material

Supplementary Information

## Figures and Tables

**Table 1:** Characteristic of the samples print on the microarrays.

**Number**	**Sample name**	**Source**	**Concentration**	**Purpose**
①	lipopolysaccharides	*E. coli* (Sigma)	1 mg mL^−1^	PAMP
②	peptidoglycan	*S. aureus* (Sigma)	1 mg mL^−1^	PAMP
③	yeast glucan	*S. cerevisiae* (Sigma)	1 mg mL^−1^	PAMP
④	β-1,3-glucan	*Euglena gracilis* (Sigma)	1 mg mL^−1^	PAMP
⑤	mannan	*S. cerevisiae* (Sigma)	1 mg mL^−1^	PAMP
⑥	lipoteichoic acids	*S. aureus* (Sigma)	1 mg mL^−1^	PAMP
⑦	CpG ODN	Digested from our constructed plasmid	20 μg mL^−1^	PAMP
⑧	poly I:C	Sigma	1 mg mL^−1^	PAMP
⑨	rabbit anti-rat IgG	rabbit	0.1 mg mL^−1^	Positive control
⑩	PBS-glycerol	–	40% (v/v)	Negative control

**Table 2:** Strategy of mutagenesis in the present study.

**Mutation No.**	**M1**	**M2**	**M3**	**M4**	**M5**	**M6**	**M7**	**M8**
Domain	CRD1	CRD2	CRD3	CRD1	CRD1	CRD2	CRD3	CRD2
Motif	Y P T	E P D	E P N	Y P T	Y P T	E P D	E P N	E P D
Target	Y S T	E S D	E S N	F P T	E P D	E P N	E P D	Q P D
