# Supplementary material for: CfLec-3 from scallop: an entrance to non-self recognition mechanism of invertebrate C-type lectin
Source: Sci Rep. 2015 May 15;5:10068. doi: 10.1038/srep10068 (PMC4432315; doi:10.1038/srep10068)
Supplement: Supplementary Information [file srep10068-s1.pdf]

CfLec-3 from scallop: an entrance to non-self recognition mechanism of invertebrates C-type lectin

Jialong Yang [1, 2, *], Mengmeng Huang [1, 2, *], Huan Zhang [1], Lingling Wang [1], Hao Wang [1], Leilei Wang [1, 2], Limei Qiu [1], Linsheng Song [1]

[1]Key laboratory of Experimental Marine Biology, Institute of Oceanology, Chinese Academy of Sciences, Qingdao, China

[2]University of Chinese Academy of Sciences, Beijing, China

*: Jialong Yang and Mengmeng Huang contribute equally to this study.

Correspondence to: Dr. Lingling Wang & Dr. Linsheng Song

Institute of Oceanology

Chinese Academy of Sciences

7 Nanhai Rd., Qingdao 266071, China

Tel: +86-532-82898552, Fax: +86-532-82880645

E-mail: wanglingling@qdio.ac.cn (LW); lshsong@ms.qdio.ac.cn (LS)

rCfLec-3

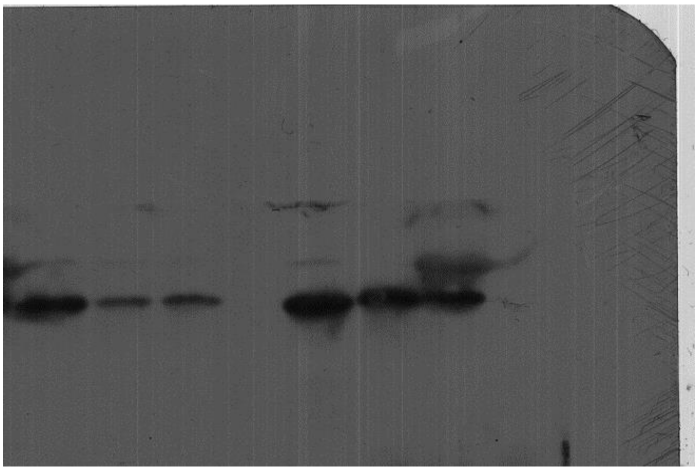

rTrx

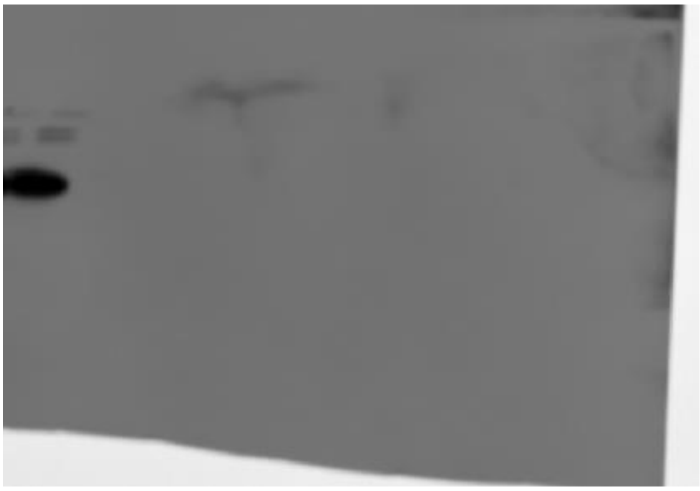

Figure S1. Original blot for figure 2c.

Table S1.The list of primers used in the present study

Primer	Sequence (5'—3')
Oligo(dT)-adaptor	GGCCACGCGTCGACTAGTACT$_{17}$
CfLec-3 RTF (forward)	GCAGAACAGACCTTCATAAATACC
CfLec-3 RTR (reverse)	ACCATATAATGTCCAGCCCA
β-actin AF (forward)	CAAACAGCAGCCTCCTCGT
β-actin AR (reverse)	CTGGGCACCTGAACCTTTCGTT
CfLec-3 REF (forward)	GGATCCGACACCGTTGATGTTGGTTGTGA
CfLec-3 RER (reverse)	GCGGCCGCTCAGTTCAGTCCCGTCCACCAAG
CRD1 REF (forward)	GGATCCGATGTTGGTTGTGACCCTGGTTGGTA
CRD1 RER (reverse)	GCGGCCGCTCAAACGTCCAGTTCACACACATATCCG
CRD2 REF (forward)	GGATCCGTCGGTGGATGTCCCTTCGGAT
CRD2 RER (reverse)	GCGGCCGCTCAGCCAAGACGACACGCAAAGTTC
CRD3 REF (forward)	GGATCCTACATGGGATGCAATGGCTGGACTC
CRD3 RER (reverse)	GCGGCCGCTCACCCTGTATATTCACAAATAGCTGGT
Mutation 1F (forward)	CAATTGCATGGGAACAATATTCTACCAGTAACTCAAAGGACTCG
Mutation 1R (reverse)	CGAGTCCTTTGAGTTACTGGTAGAATATTGTTCCCATGCAATTG
Mutation 2F (forward)	CCTGGGCTAAGGAATCGGACCACATAAACAACAACG
Mutation 2R (reverse)	CGTTGTTGTTTATGTGGTCCGATTCCTTAGCCCAGG
Mutation 3F (forward)	CGGTGGAATCAGGAGTCCAACTCGTGGTTTG
Mutation 3R (reverse)	CAAACCACGAGTTGGACTCCTGATTCCACCG
Mutation 4F (forward)	CAATTGCATGGGAACAATTTCCTACCAGTAACTCAAAGGACTC
Mutation 4R (reverse)	GAGTCCTTTGAGTTACTGGTAGGAAATTGTTCCCATGCAATTG
Mutation 5F (forward)	CAATTGCATGGGAACAAGAACCGGACAGTAACTCAAAGGAC
Mutation 5R (reverse)	GTCCTTTGAGTTACTGTCCGGTTCTTGTTCCCATGCAATTG
Mutation 6F (forward)	CCTGGGCTAAGGAACCGAACCACATAAACAACAACG
Mutation 6R (reverse)	CGTTGTTGTTTATGTGGTTCGGTTCCTTAGCCCAGG
Mutation 7F (forward)	CGGTGGAATCAGGAGCCCGACTCGTGGTTTG
Mutation 7R (reverse)	CAAACCACGAGTCGGGCTCCTGATTCCACCG
Mutation 8F (forward)	CCTGGGCTAAGCAACCGGACCACATAAACAACAACG
Mutation 8R (reverse)	CGTTGTTGTTTATGTGGTCCGGTTGCTTAGCCCAGG
